# Is Immune Suppression Involved in the Ischemic Stroke? A Study Based on Computational Biology

**DOI:** 10.3389/fnagi.2022.830494

**Published:** 2022-02-10

**Authors:** Xin Wang, Qian Wang, Kun Wang, Qingbin Ni, Hu Li, Zhiqiang Su, Yuzhen Xu

**Affiliations:** ^1^Department of Neurology, First Affiliated Hospital of Harbin Medical University, Harbin, China; ^2^Postdoctoral Workstation, Taian City Central Hospital, Taian, China; ^3^Department of Rehabilitation, The Second Affiliated Hospital of Shandong First Medical University, Taian, China

**Keywords:** immunosuppression, ischemic stroke, genes, transcription factors, bioinformatics

## Abstract

**Objective:**

To identify the genetic mechanisms of immunosuppression-related genes implicated in ischemic stroke.

**Background:**

A better understanding of immune-related genes (IGs) involved in the pathophysiology of ischemic stroke may help identify drug targets beneficial for immunomodulatory approaches and reducing stroke-induced immunosuppression complications.

**Methods:**

Two datasets related to ischemic stroke were downloaded from the GEO database. Immunosuppression-associated genes were obtained from three databases (i.e., DisGeNET, HisgAtlas, and Drugbank). The CIBERSORT algorithm was used to calculate the mean proportions of 22 immune-infiltrating cells in the stroke samples. Differential gene expression analysis was performed to identify the differentially expressed genes (DEGs) involved in stroke. Immunosuppression-related crosstalk genes were identified as the overlapping genes between ischemic stroke-DEGs and IGs. Feature selection was performed using the Boruta algorithm and a classifier model was constructed to evaluate the prediction accuracy of the obtained immunosuppression-related crosstalk genes. Functional enrichment analysis, gene-transcriptional factor and gene-drug interaction networks were constructed.

**Results:**

Twenty two immune cell subsets were identified in stroke, where resting CD4 T memory cells were significantly downregulated while M0 macrophages were significantly upregulated. By overlapping the 54 crosstalk genes obtained by feature selection with ischemic stroke-related genes obtained from the DisGenet database, 17 potentially most valuable immunosuppression-related crosstalk genes were obtained, ARG1, CD36, FCN1, GRN, IL7R, JAK2, MAFB, MMP9, PTEN, STAT3, STAT5A, THBS1, TLR2, TLR4, TLR7, TNFSF10, and VASP. Regulatory transcriptional factors targeting key immunosuppression-related crosstalk genes in stroke included STAT3, SPI1, CEPBD, SP1, TP53, NFIL3, STAT1, HIF1A, and JUN. In addition, signaling pathways enriched by the crosstalk genes, including PD-L1 expression and PD-1 checkpoint pathway, NF-kappa B signaling, IL-17 signaling, TNF signaling, and NOD-like receptor signaling, were also identified.

**Conclusion:**

Putative crosstalk genes that link immunosuppression and ischemic stroke were identified using bioinformatics analysis and machine learning approaches. These may be regarded as potential therapeutic targets for ischemic stroke.

## Introduction

Ischemic stroke is the second most common cause of mortality worldwide and imposes a tremendous healthcare burden owing to significant disability. Aging populations are likely to further compound the gravity of the burden imposed by stroke (Katan and Luft, 2018). The prevention and clinical management of stroke assume critical importance. As currently widely applied modalities are limited in their clinical efficacy, a need for the advent of novel approaches is recognized. Molecular mechanisms involved in the pathophysiology of stroke resulting from cerebral ischemia and consequent brain tissue damage, involve immune activation, which leads to a host of both neuroprotective and neuro-toxic effects (Iadecola and Anrather, 2011; Iadecola et al., 2020). Further, stroke is frequently followed by post-stroke infection, which results from systemic immunosuppression that occurs after stroke and is associated with a worse prognosis (Shi et al., 2018). This occurs as a bi-directional brain-immune system interaction, when catecholamines and glucocorticoids are produced by an activated HPA-axis in attempts to limit local inflammation, which leads to natural killer T-cell (NKT) and T-cell activation, alongside reactive oxygen species production (Shim and Wong, 2016). Immune-modulatory approaches involving T-cell transfer, natural killer T-cell (NKT) activators and interferon-gamma (IFN-γ) have shown benefit in reducing post-stroke immunosuppression (Wong et al., 2011; Liu et al., 2017). Immunomodulatory approaches that target multiple elements of the immune system are now recognized as the most promising directions in stroke and its complication management (Fu et al., 2015). Several established drugs such as Azithromycin and Metformin have shown neuroprotective action in stroke via modulation of innate immune responses (Amantea and Giacinto, 2016) and the repurposing of such drugs may offer therapeutic potential, thus underscoring the importance of uncovering immunosuppression mechanisms in stroke.

However, at present, the immune mechanisms implicated in stroke and stroke-associated systemic immunosuppression are still poorly understood. A comprehensive understanding of the immune mechanisms relevant to stroke has the potential to identify target genes and functional pathways, which can direct therapeutic interventions including drug development and repurposing. Integrated bioinformatics analysis of gene expression or transcriptomic datasets associated with disease can leverage higher scales of data to generate valuable insights. Gene expression data can also enable *in silico* identification of heterogeneous cell populations within a sample, including immune activate cell subsets (Newman et al., 2015). Furthermore, the application of machine learning-based feature selection algorithms can highlight potentially the most important genes linked to a disease or condition (Kursa, 2014). Therefore, in the current study, we aimed to utilize bioinformatics tools combined with feature selection to perform comprehensive secondary analysis of multiple transcriptomic datasets in stroke to reveal key immunosuppression related genes implicated in its pathogenesis. This approach could help highlight genes and pathways of high value for clinical and therapeutic translation for managing stroke and related immunosuppression.

## Materials and Methods

### Gene Expression Datasets in Stroke and Immunosuppression-Related Gene Data

Two gene-expression microarray datasets pertaining to ischemic stroke were identified and downloaded from the NCBI Gene Expression Omnibus (GEO) database,^1^ GSE16561 and GSE22255. GSE16561 included peripheral blood samples from 39 stroke patients and 24 controls in a total of 63 samples analyzed using the “Illumina HumanRef-8 v3.0 Expression BeadChip” array (Barr et al., 2010) and GSE22255 included peripheral blood mononuclear cells from 20 stroke patients and 20 controls in a total of 40 samples analyzed using the “Affymetrix Human Genome U133 Plus 2.0” array (Krug et al., 2012).

Next, immunosuppression-related genes were downloaded from DisGenet^2^ and HisgAtlas.^3^ In addition, drugs related to immunosuppressive agents were sourced from Drugbank.^4^ Here, 311 immunosuppressive drugs were identified and the relevant immunosuppressive related genes were downloaded. The immunosuppression-related genes obtained from these three databases (DisGeNET, HisgAtlas, and Drugbank) were merged to obtain a total of 1,332 genes, and the expression profiles of these specific immunosuppression-related genes in the GSE16561 and GSE22255 datasets were extracted.

### Analysis of Immune-Cell Subsets Enriched in Stroke

The expression levels of immunosuppression-related genes in stroke were normalized and the “CIBERSORT” deconvolution algorithm (Newman et al., 2019) in the R statistical environment was applied to predict the infiltrating immune cell subsets that are highly related to stroke. The expression matrix of immunosuppression-related genes in stoke was used as the input, with 1,000 permutations and quantile normalization. A heatmap was then constructed to display immune cell subsets in each sample.

### Differential Gene Expression Analysis and Functional Enrichment Analysis

The downloaded GSE16561 and GSE22255 gene expression datasets were subjected to preprocessing and filtration. Differential gene expression was performed using the R package “limma” with a *P*-value < 0.05 and logFC change > 0.58 set as thresholds to determine differentially expressed genes (DEGs) and volcano plots were plotted to display the results. Gene ontology (GO) functional profiles of the significant differentially expressed genes from these two data sets were clustered based on similarity using the “compareCluster_go” function of the “clusterProfiler” R package (Yu et al., 2012). Among these, pathways with *P*-value < 0.05 were considered as significantly enriched.

### Protein-Protein Interaction Network

Protein-protein interaction (PPI) data was downloaded from multiple databases including PBIND (currently offline), BioGRID),^5^ MINT,^6^ HPRD,^7^ IntAct^8^ and OPHID^9^ and were integrated and the PPI relationship pairs of the immunosuppression-related DEGs were extracted from the integrated PPI data. A PPI network was constructed using Cytoscape and topological properties were analyzed using the “networkanalyzer” tool (Gustavsen et al., 2019). Gene nodes with the highest degree (indicating network connectivity) were considered to be of high functional importance in stroke and the top 30 genes were thus annotated as important stroke-related genes.

### Identification of Immunosuppression-Related Crosstalk Genes and Protein-Protein Interaction Network

Immunosuppression-related genes were downloaded from the InnateDB^10^ database and intersecting DEGs in the GSE16561 and GSE22255 datasets were identified among these, merged with the earlier identified immunosuppression-related DEGs, and labeled as immunosuppression-related crosstalk genes. PPI relationship pairs for these genes were extracted and a crosstalk gene PPI network was constructed using Cytoscape.

### Feature Selection of Most Important Immunosuppression-Related Crosstalk Genes

The immunosuppression-related crosstalk genes were considered as stroke-related features and the Boruta algorithm was applied using the R package “Boruta” (Kursa and Rudnicki, 2010) to select the most representative features. Boruta algorithm is a supervised classification feature selection method that is based on random forest and identifies all relevant features for classification task. The central concept in this algorithm is the iterative comparison of the actual predictor variables to generated shadow variables using random forest, thus sequentially identifying the variables relevant to classification. In the first step, the gene expression profiles of these crosstalk genes in the GSE16561 dataset were extracted. In the second step, the Boruta algorithm was applied to group features based on the category of each sample, with a *p*-value < 0.01 and the default maximum run at 100 times. The outcome grouped the crosstalk gene features into three categories: tentative temporary/pending features (not enough to accept or reject), confirmed features, and rejected features. For the tentative features, the “TentativeRoughFix” function was applied. Assuming that the Boruta algorithm might have preference errors, a traditional recursive feature elimination algorithm (RFE) was also applied to perform feature screening on the extracted immune gene expression values and the outcomes were compared.

### Classifier Models Based on Crosstalk Immunosuppression-Related Crosstalk Genes

First of all, considering that the expression values in the GSE16561 and GSE22255 data sets were of different magnitudes and the GSE16561 data was normalized but GSE22255 was not, the GSE22255 data was normalized so that the two datasets were consistent. Next, the crosstalk genes obtained using the Boruta algorithm were verified using the two standardized data sets, and the “k nearest neighbor” method was applied to fill in missing (NA) values. The Decision tree, AdaBoost, and SVM algorithms were used to build classifier models. Here, randomly selected samples from the GSE16561 and GSE22255 datasets were used as a training set. Randomly selected 70% samples from the GSE16561 series were set as a test set and the crosstalk gene expression data from the GSE22255 data set was used as the verification set. To predict the accuracy of the classifier models, the test set data was input into the trained classifier for verification, and the receiver operating curves (ROC) curves of the prediction results were plotted using the “pROC” R package. Next, 1,159 genes related to ischemic stroke were downloaded from the “DisGenet” database. The crosstalk immunosuppression-related genes obtained were intersected with these to identify the potentially most valuable classifier genes and the accuracy of the selected genes was examined.

### Pathway Analysis of the Immunosuppression-Related Crosstalk Genes

The crosstalk genes were subjected to KEGG pathway analysis and the top 30 enriched pathways were represented as key pathways of interest.

### Crosstalk Gene-Transcription Factor Network Analysis

Transcription factors (TF) and target gene pairs were downloaded from the TRRUST^11^ and ORTI^12^ databases and the data were pooled. The TF-target gene relationship pairs of the crosstalk genes were extracted the TF-crosstalk gene network was plotted in Cytoscape. The topological properties of the top 10 genes ranked by outdegree were analyzed to gain insights into the transcriptional regulation of immunosuppression-related genes in stroke pathogenesis.

### Biological Pathways Analysis Enriched by the Transcription Regulatory Crosstalk Genes

The TF-target gene relationship pairs were extracted from the transcriptional regulatory network, and these genes were divided into two gene sets, genes that were up-regulated and those that were down-regulated. The “enrichGO()” function of the “clusterprofiler” R package was applied to separately analyze biological pathways enriched in these two gene sets, with a *p*-value < 0.05. The top 30 enriched biological pathways were reported as those being most significant pathways.

### Crosstalk Gene- Drug Target Network Analysis

To identify drugs that may be potentially relevant to stroke by immune modulation, gene-drug interaction pair data was downloaded from the DIGDB database.^13^ Drugs related to the most significant crosstalk genes identified earlier to construct a gene-drug network.

## Results

### Immune-Cell Subsets Enriched in Stroke

**FIGURE 1:**
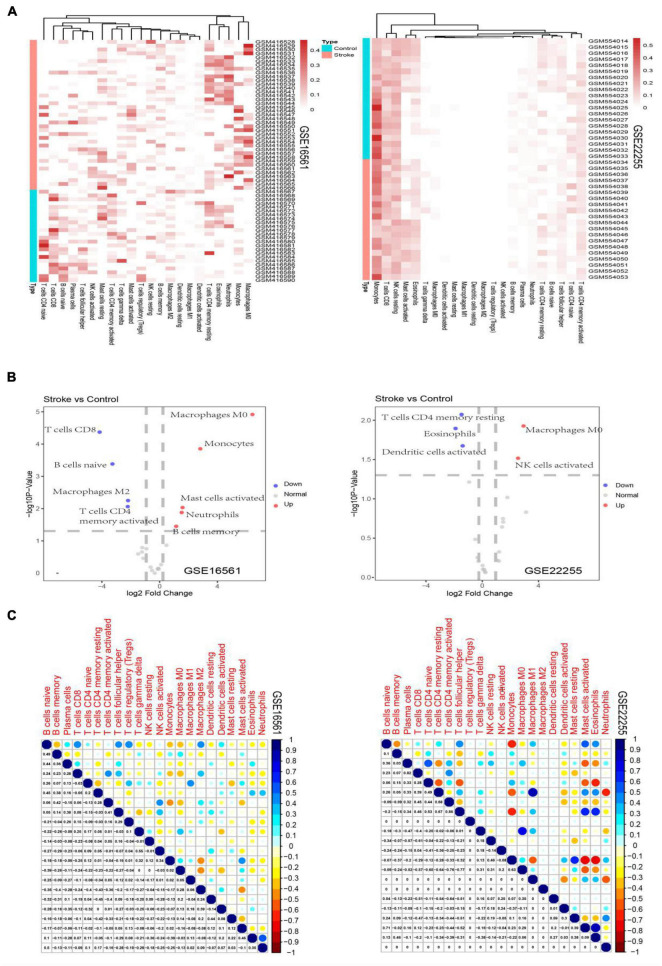
Heatmaps depicting the proportion of immune cell subtypes in the GSE16561 and the GSE22255 datasets **(A)**. Volcano plots depict the log2fold change of significantly up-regulated and down-regulated cellular subsets in the GSE16561 and the GSE22255 datasets **(B)**. Correlogram plots depicting the correlations between immune cell subtypes **(C)**.

The derived cellular component subsets in the samples are displayed as heatmaps and volcano plots (Figure 1) and significant differences were noted in the immune cell components between the stroke and normal samples. As samples were not labeled correctly, filtering was applied. Thereafter, GSE16561 retained 48 samples while GSE22255 retained 24 samples. Two subtypes of immune cells, resting CD4 memory T cells and M0 Macrophages were significantly different between the cases and controls. CD4 memory T cells were significantly down-regulated in stroke whereas M0 Macrophages were noted as significantly up-regulated.

### Differentially Expressed Genes and Functional Enrichment Analysis

**FIGURE 2:**
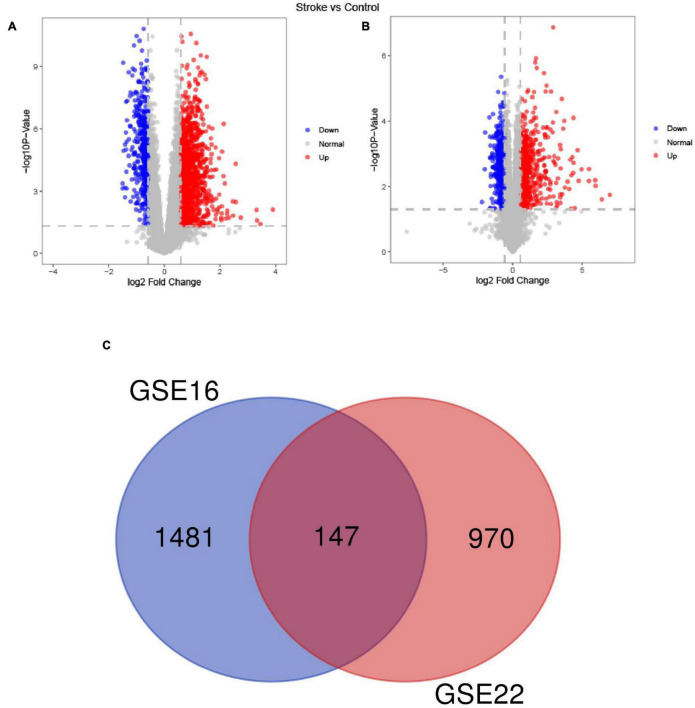
A Venn diagram of the up- and down-regulated DEGs in the GSE16561 **(A)** and GSE22255 **(B)** datasets and the Venn diagram depicts DEGs shared by the GSE16561 and GSE22255 series of samples **(C)**.

**FIGURE 3:**
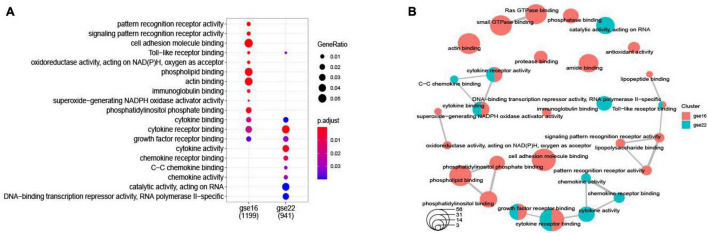
A dotplot of the GO function enrichment nodes of DEGs in the GSE16561 and GSE22255 datasets **(A)**; an emapplot of the GO function enrichment nodes in GSE16561 and GSE22255 datasets **(B)**.

Among the DEGs determined in the two datasets, 147 DEGs were shared (Figure 2). The top 10 most highly significantly enriched pathways are depicted in Figure 3. Nodes representing regulatory pathways that control cell growth and division were found overlapping amongst the results from the two datasets. Other sets of functional pathways enriched in the DEGs varied between the two datasets, plausibly owing to sample heterogeneity.

### Protein-Protein Interaction Network in Stroke

**FIGURE 4:**
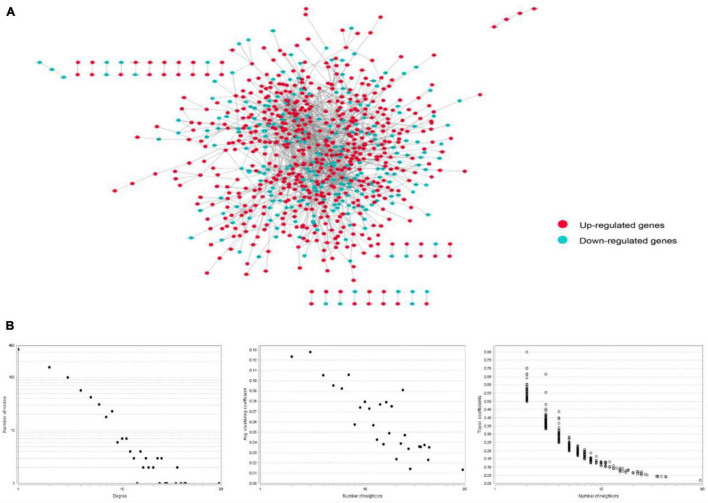
**(A)** The protein-protein interaction network consisted by integrating the differentially expressed genes in two datasets. There are 1,460 relational pairs and 821 nodes in the network. **(B)** The various statistical indicators in the PPI network shown in **(A)**.

The PPI network of integrated DEGs of the two datasets is represented in Figure 4. The network consisted of 1,460 PPI pairs and 821 nodes. The gene nodes with topmost degree ranks in the PPI network included GRB2, MAPK1, TP53, FYN, PXN and Table 1 presents the top 10 gene nodes in the network.

**TABLE 1:** Top 30 ranked genes in the PPI network of integrated DEGs.

Symbol	Degree	Average Shortest Path Length	Betweenness Centrality	Closeness Centrality	Clustering Coefficient	Topological Coefficient
GRB2	86	2.76315789	0.24334414	0.36190476	0.01340629	0.02064727
MAPK1	41	2.86973684	0.10984015	0.34846401	0.03536585	0.03951394
TP53	40	3.05921053	0.07761154	0.32688172	0.02307692	0.04166667
FYN	37	2.95263158	0.08285592	0.33868093	0.03753754	0.0418483
PXN	34	2.88552632	0.10707404	0.34655723	0.04278075	0.04506822
CREBBP	34	3.25	0.06912861	0.30769231	0.0285205	0.04338757
CASP3	33	3.02105263	0.09005441	0.33101045	0.03598485	0.04453627
YWHAG	27	3.30921053	0.04671586	0.30218688	0.01424501	0.05149051
SMAD3	26	3.19736842	0.06550871	0.3127572	0.03384615	0.05560704
LYN	24	3.18552632	0.03191512	0.31391987	0.05072464	0.06045279
JUN	24	3.23157895	0.03011403	0.30944625	0.0615942	0.06934932
MAPK14	24	3.11184211	0.0477154	0.32135307	0.02898551	0.05197368
SP1	23	2.91710526	0.0673405	0.34280559	0.09090909	0.06418972
MAPK3	22	3.14473684	0.02389596	0.31799163	0.06060606	0.07029309
STAT3	22	3.26315789	0.03607932	0.30645161	0.03896104	0.07188161
YWHAH	22	3.375	0.03049526	0.2962963	0.01731602	0.05947324
PIK3R1	20	3.39605263	0.03433461	0.29445951	0.01578947	0.069
HSP90AB1	20	3.11184211	0.04933295	0.32135307	0.03157895	0.0642132
HSPA8	18	3.02368421	0.05323372	0.33072237	0.08496732	0.0790687
PRKCD	18	3.28289474	0.02483384	0.30460922	0.06535948	0.08389262
STAT1	17	3.28947368	0.04105675	0.304	0.01470588	0.0681606
PRKDC	17	3.09210526	0.0327038	0.32340426	0.08088235	0.07782405
HSPA1A	17	3.36710526	0.03019882	0.29699101	0.05147059	0.07769145
VCL	16	3.48157895	0.02689397	0.287226	0.03333333	0.07515823
PAK2	16	3.14342105	0.02459102	0.31812474	0.125	0.09440104
TNFRSF1A	15	3.47368421	0.02722882	0.28787879	0.11428571	0.09318996
SNCA	15	3.40263158	0.03034559	0.29389018	0.02857143	0.08539945
CDC42	15	3.53289474	0.02840219	0.283054	0.00952381	0.07843137
LRIF1	15	3.81842105	0.02597249	0.26188835	0	0.07083333
VIM	14	3.30526316	0.02247123	0.30254777	0.07692308	0.09307359

### Immunosuppression-Related Crosstalk Genes

**FIGURE 5:**
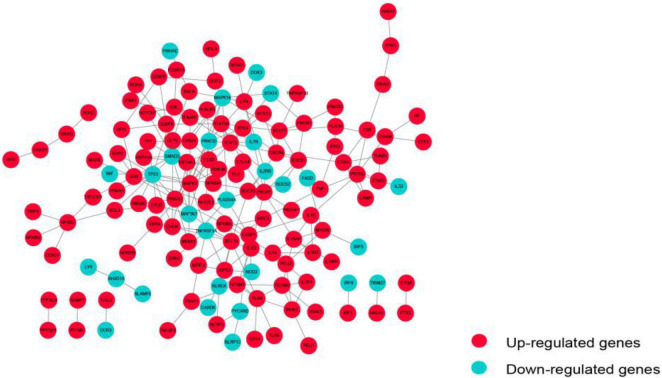
The PPI network of immunosuppression-related crosstalk genes.

The PPI network of these immunosuppression-related crosstalk genes comprising of 213 PPI pairs and 140 nodes is depicted in Figure 5. There are 213 international pairs and 140 nodes in the network.

### Feature Selection From Immunosuppression-Related Crosstalk Genes

**FIGURE 6:**
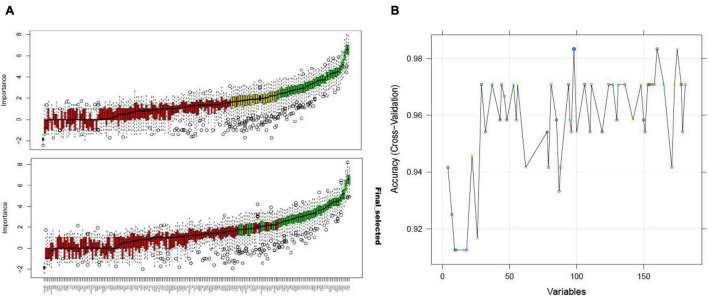
The Boruta algorithm feature selection results of the GSE16561 dataset showing the results before and after the “TentativeRoughFix” function to remove undetermined features **(A)**; accuracy score of the number of feature selections using the RFE algorithm for the GSE16561 dataset **(B)**.

Using the Boruta algorithm, 54 immune-related crosstalk gene features were selected as representative features (Figure 6A). 98 feature genes were selected by the RFE algorithm (Figure 6B), which included all 54 genes selected using the Boruta algorithm, validating its good performance.

### Classifier Models Based on Immunosuppression-Related Crosstalk Genes

**FIGURE 7:**
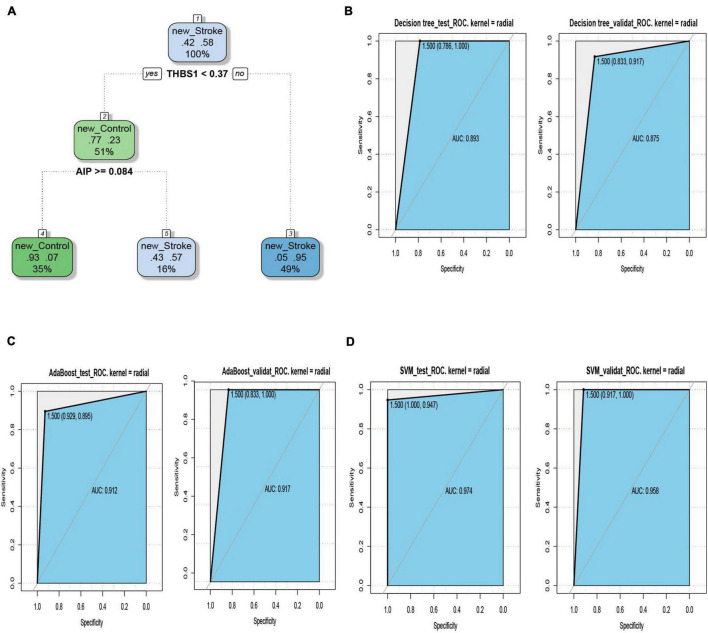
The decision tree diagram of the classifier model **(A)**. ROC curves of the test and validation sets of three classifiers; Decision tree **(B)**, AdaBoost **(C)** and SVM **(D)**.

The ROC curves pertaining to the dataset used to evaluate the classifier models are represented in Figure 7. The SVM classifier showed the best performance. The AUC values for the test set and validation set were 0.974 and 0.958, respectively, indicating the excellent performance of the classifier model to predict stroke patients and normal samples (Table 2). Among the 54 crosstalk immune-related genes, 17 genes were noted in the DisGenet database including, “ARG1” “CD36” “FCN1” “GRN” “IL7R” “JAK2” “MAFB” “MMP9” “PTEN” “STAT3” “STAT5A” “THBS1” “TLR2” “TLR4” “TLR7” “TNFSF10” “VASP.”

**TABLE 2:** Test results of 3 classifiers.

Number	Dataset	Decision tree	AdaBoost	SVM
1	Test set	0.893	0.912	0.974
2	validation set	0.875	0.917	0.958

**FIGURE 8:**
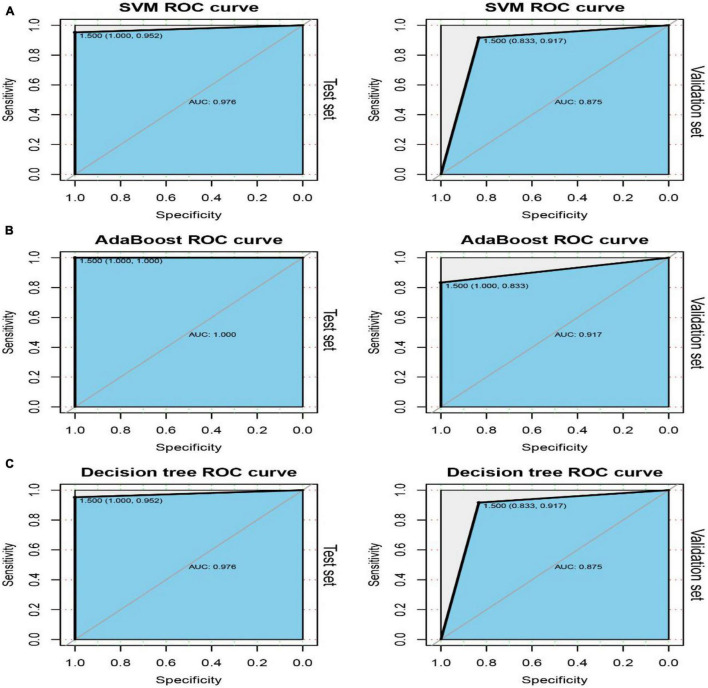
ROC curves of the test and validation sets of three classifiers: SVM **(A)**, AdaBoost **(B)** and Decision tree **(C)**.

Among the 54 crosstalk immune-related genes, 17 genes were noted in the DisGenet database including, “ARG1” “CD36” “FCN1” “GRN” “IL7R” “JAK2” “MAFB” “MMP9” “PTEN” “STAT3” “STAT5A” “THBS1” “TLR2” “TLR4” “TLR7” “TNFSF10” “VASP.” Another classifier model was constructed using these 17 genes and the ROC curves were plotted (Figure 8).

### Pathway Analysis of the Immunosuppression-Related Crosstalk Genes

**FIGURE 9:**
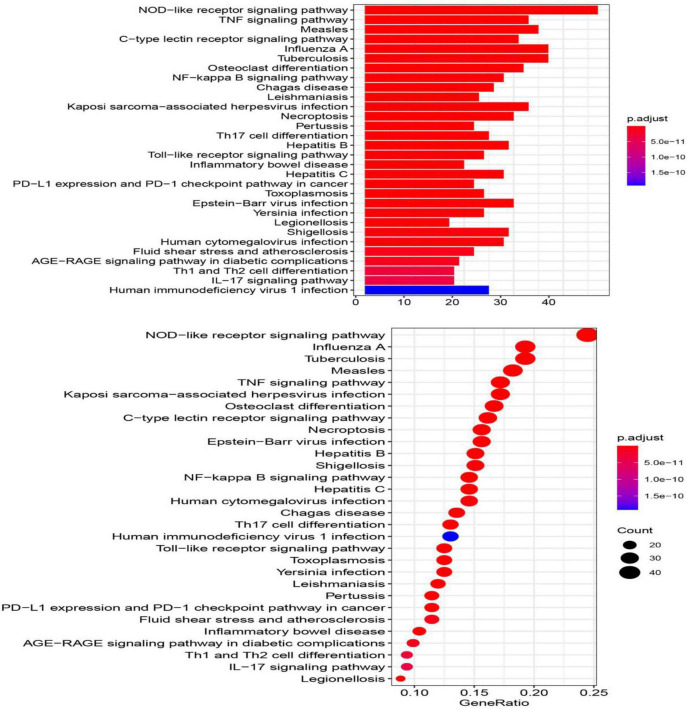
The top 30 KEGG pathways enriched in the immune-related crosstalk genes.

The top 30 KEGG pathways enriched in the immune-related crosstalk genes are represented in a barplot and dotplot (Figure 9). NOD-like receptor signaling pathway showed the highest significance and gene ratio values. TNF-signaling, Measles, Tuberculosis, C-type leptin receptor signaling and osteoclast differentiation were among the topmost enriched pathways.

### Immunosuppression-Related Crosstalk Gene-Transcription Factor Network Analysis

**FIGURE 10:**
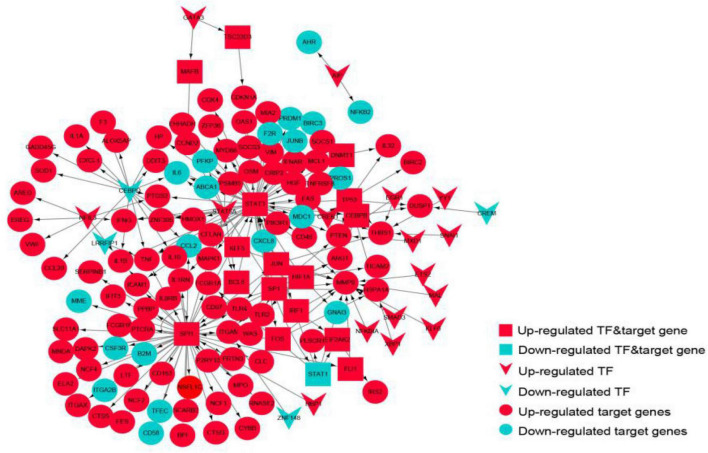
The transcriptional regulatory network of the crosstalk genes. Red represents up-regulated genes, blue represents down-regulated genes, circles represent target genes, arrows represent transcription factors, and rectangles represent targets.

The TF-target gene network consisted of 146 nodes and 208 TF-target gene pairs (Figure 10). The topological properties of top 10 genes in the network ranked by outdegree is presented in Table 3. STAT3, SPI1, CEBPD, STAT5A, and SP1 were ranked the highest in this network.

**TABLE 3:** Top 10 ranked genes in the transcription factor-crosstalk gene regulatory network.

SYMBOL	Outdegree	Average Shortest Path Length	Betweenness Centrality	Closeness Centrality	Clustering Coefficient	Expression_type
STAT3	56	1.83333333	0.04281957	0.54545455	0.00526008	*Up*
SPI1	50	1.65740741	0.0308494	0.60335196	0.00580552	*Up*
CEBPD	16	1.05882353	0	0.94444444	0	*Down*
STAT5A	13	2.76576577	0	0.36156352	0	*Up*
SP1	10	2	0.00361269	0.5	0.05454545	*Up*
TP53	7	2.72222222	0.00152332	0.36734694	0.02380952	*Up*
NFIL3	6	1	0	1	0	*Up*
STAT1	5	2.74074074	0.00134852	0.36486486	0.05	*Down*
HIF1A	5	2.75	0.0015483	0.36363636	0	*Up*
JUN	4	2.58333333	0.00621816	0.38709677	0.15	*Up*

### Biological Pathways Analysis Enriched by the Transcription Regulatory Immunosuppression-Related Crosstalk Genes

**FIGURE 11:**
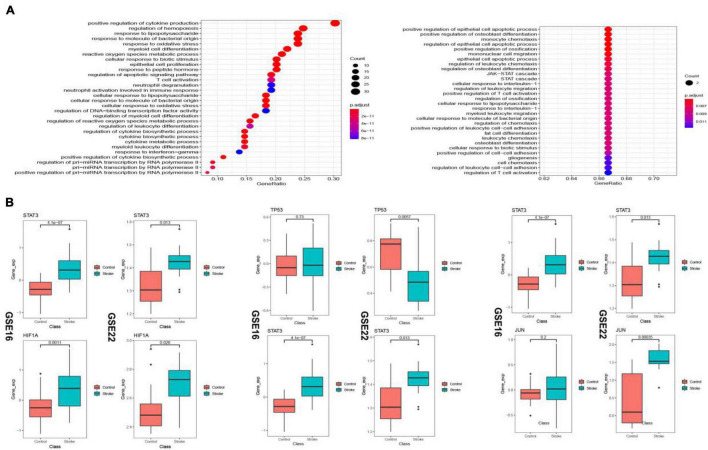
Biological pathways (BP) enriched in the up- and down-regulated transcriptional regulatory immunosuppression-related crosstalk genes **(A)**. Expression levels of key transcription factors and corresponding target crosstalk genes in the stroke and control samples in the two datasets **(B)**.

The up-regulated crosstalk immunosuppression-related genes were mainly enriched in biological pathways related to cell growth and proliferation, and energy metabolism, while the down-regulated genes were mainly enriched in positive cell apoptosis. The biological pathways related to cell differentiation and migration indicated that up- and down-regulated genes function together to promote the occurrence and development of stroke (Figure 11A). Expression levels of 10 key genes screened in the transcriptional regulatory network showed that the STAT3 gene and HIF1A gene showed similar transcriptional regulatory relationship in both the datasets, whereas TP53 as a transcription factor and its target gene JUN did not consistency in the expression pattern (Figure 11B) suggesting immune type heterogeneity in stroke.

### Drugs Targeted by Immunosuppression-Related Crosstalk Genes

**FIGURE 12:**
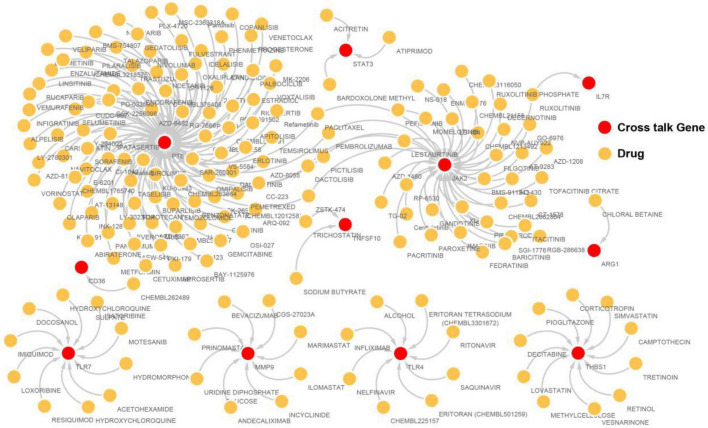
The immunosuppression-related crosstalk genes-target drugs network.

Drugs related to the 17 immunosuppression-related cross talk genes were screened and 11 genes were found to have target drugs. A gene-drug network is presented in Figure 12. The maximum numbers of drug targets were noted for the PTEN gene, followed by the JAK2 gene.

## Discussion

The current study provides insights into the immune landscape of stroke via a comprehensive bioinformatic analysis of key immunosuppression-related genes underpinning the pathogenesis of stroke. Using a machine learning approach, a small number of 17 potentially most relevant immunosuppression-related crosstalk gene features were identified, which showed high performance accuracy in classifying stroke from healthy samples suggesting their potential value in molecular diagnosis and as biological targets.

The initial findings showed that CD4 memory T cells were significantly downregulated while M0 Macrophages were upregulated in stroke. CD4 T cell infiltration has been implicated in stroke-associated neuroinflammation (Yilmaz et al., 2006; Gu et al., 2013) and CD4 cell deficit has shown beneficial effects by reducing infarction. The seemingly contrasting finding from our study may be accounted for by the temporal sequence of T cell infiltration in ischemic stroke, whereby animal models of stroke have shown that the peak of T cell infiltration appears after 3–5 days in transient ischemia, while other report CD4 T cells showed a rise beginning from 7 to 30 days after stroke (Gelderblom et al., 2009; Shichita et al., 2009; Stubbe et al., 2012). The samples used in the present study included blood collected within 24 h of symptom onset (Barr et al., 2010) and whereas the exact duration was not specified in one dataset (Krug et al., 2012), therefore, the temporal variation in immune cell infiltration could not be assessed. Macrophages and microglia play key roles in mediating tissue injury during the acute phase of ischemic stroke (Mabuchi et al., 2000; Hu et al., 2012). Infiltration of blood monocytes through a breached blood-brain barrier is an important early event in the neuroinflammation of post-stroke brain tissue (Mo et al., 2013). C-C chemokine receptor 2 (CCR2) + Mo/MΦ have been found to spread through the brain tissue in the early phase of stroke and have been implicated in both early stage inflammation and later functional recovery (Garcia-Bonilla et al., 2016; Fang et al., 2018; Pedragosa et al., 2020).

The PPI network of DEGs in stroke indicated that the top genes of relevance were GRB2, MAPK1, TP53, FYN, and PXN. The involvement of these genes in stroke pathology is largely supported by experimental data. GRB2 encodes for a protein that binds the epidermal growth factor receptor leading to downstream mitogen-activated protein kinase (MAPK) signaling and is implicated in the development of atherosclerotic lesions (Proctor et al., 2007). GRB2 has been implicated in neuronal autophagy in stroke neuropathology via the suppression of the Akt/mTOR pathway (Luo et al., 2020). p38 mitogen-activated protein kinase (MAPK) signaling is a key mediator of inflammation, being a critical mediator of cell responses to cytokines and MAPK signaling has been widely implicated in the pathogenesis of stroke (Sun and Nan, 2016), although the specific upstream and downstream mechanisms remain unclarified, and different brain tissue types are marked by varying MAPK signaling patterns after ischemic stroke (Sawe et al., 2008). Animal data have shown that inhibition of p38 MAPKα starting in the early period after stroke led to improvement in motor and somatosensory function (Alam et al., 2020), whereas others have reported that MAPK-1 signaling plays an endogenous protective role in stroke and its inhibition accelerated stroke-induced injury via its anti-apoptotic and anti-inflammatory actions (Liu et al., 2014). MAPK inhibitors are emerging as promising anti-inflammatory agents for acute stages of brain inflammation (Kaminska et al., 2009) including cerebrovascular disorders (Ansar et al., 2013).

TP53 polymorphism has been found associated with the prognosis of post-stroke functional recovery (Gomez-Sanchez et al., 2011) and neuroprotection after ischemia (Ramos-Araque et al., 2018). Furthermore, methylation of TP53 promoter has been associated with ischemic stroke (Wei et al., 2019). A recent bioinformatic study investigating ischemic stroke-related targets for the herbal medication *Calculus Bovis* reported MAPK1 and TP53 among the top target genes (Liu et al., 2021).

FYN is a type of Src Family Kinase (SFK) gene, that code for a non-receptor protein tyrosine kinase and SFK inhibitors have shown protective action against ischemic stroke in animals (Takenaga et al., 2009). FYN is shown to interact with NMDAR during the ischemic response (Takagi et al., 1999) and phosphorylation of several proteins, which is implicated in cell death in stroke via reactive oxygen species generation and calcium flux (Knox and Jiang, 2015). In a rabbit, increased expression of FYN gene after ischemic stroke has been documented, and the authors suggested that GP6 induced activation of FYN initiated phosphorylation events eventually accelerating platelet adhesion (Gu et al., 2021).

Paxillin or PXN is implicated in oligodendrocyte differentiation (Yamauchi et al., 2006). Downregulation of PXN in response to treatment of ischemic stroke with two Chinese medicine derived bioactive compounds has been documented (Liu et al., 2012). Similar downregulation of PXN by Diphenyleneiodonium for the treatment of stroke has been also documented (Nagel et al., 2012) although experimental evidence describing functional pathways of PXN involvement in stroke pathology is scarce. In a concordant finding, a bioinformatics analysis of inflammation associated competing endogenous RNA network in ischemic stroke has reported FYN and PXN among the most significantly enriched modules from the hub genes (Zhang et al., 2020).

The SVM classifier showed excellent AUC values for discrimination of ischemic stroke and pathway analysis of the immunosuppression-related crosstalk genes showed TNF-signaling among the topmost enriched pathways. TNF-signaling plays pleiotropic and multiple roles in ischemic stroke, including acute inflammation and cell death during the early stages and tissue repair during the later recovery phase (Sairanen et al., 2001). The disease-related KEGG pathways enriched in the 17 key immunosuppression-related crosstalk genes including Measles and Tuberculosis pathways and osteoclast differentiation have been similarly reported as top pathways implicated in stroke (Diao and Liu, 2017). Leptin receptors are expressed by astrocytes (Schwartz and Baskin, 2013) and treatment with Leptin has shown improvements in post-stroke edema and functional recovery (Busch et al., 2010). In the transcriptional regulatory network of the immunosuppression-related crosstalk genes, STAT3 and SPI1 showed very high degrees. Activation of the JAK/STAT3 pathway in stroke is well-documented (Planas et al., 1996; Choi et al., 2003; Satriotomo et al., 2006) and its inhibition is found to significantly reduce the inflammatory process (Wu et al., 2018). In agreement with our findings, transcriptomic analysis of astrocytes has shown Stat3, Sp1, and Spi1 as the most significant transcription factors in stroke (Rakers et al., 2018).

Along with STAT3, HIF1A showed a consistent transcriptional regulatory relationship in both datasets. HIF1A or hypoxia-inducible factor 1, is implicated in cellular adaptation to hypoxia and its inhibition was found to increase mortality and infarct volume size, while its downstream genes were found to increase ischemia-induced blood-brain barrier permeability on one hand, but also induce neuroprotection on the other hand (Yan et al., 2011). HIF1A has been considered an important target for the management of stroke (Pan et al., 2021). Analysis of drug targets for the immunosuppression-related crosstalk genes showed PTEN and JAK2 as the most prominent targets. PTEN or Phosphatase and tensin homolog deleted on chromosome 10 inhibitor drugs administration after stroke is found to confer neuroprotection via improvement of axonal growth and activation of the Akt/mTOR activation (Mao et al., 2013). Small molecule PTEN inhibitors currently under investigation include BPV that has shown protective effects in experimental stroke (Mao et al., 2013) and SF1670 (Li et al., 2011). JAK2 is a part of the Janus Kinase family and are implicated in cytokine and growth factor-related signaling and JAK targeting has been proposed as a strategy for the management of multiple immune and inflammatory diseases (Schwartz et al., 2017).

The findings of the present study must be considered in light of its limitations. The included microarray datasets were of small sample size and RNA-seq datasets were not included. Large sampled transcriptome datasets with detailed metadata including time after stroke are essential to decipher the molecular events in the immune cascade following stroke. Additionally, experimental *in vitro* and *in vivo* investigations to verify the identified relevant immunosuppression-related genes in stroke were not conducted. In addition, the functional pathways involved remain to be investigated in future targeted studies. Therefore, future studies are necessary to confirm the involvement of the highlighted immune crosstalk genes and their related mechanistic pathways in ischemic stroke, in order to facilitate the clinical translation of these findings. At the same time, the strength of the current study includes the application of multiple bioinformatics and machine learning approaches to identify the most relevant immunosuppression-related genes in stroke.

## Conclusion

The present bioinformatic study utilized a comprehensive approach to analyze immunosuppression-related genes implicated in stroke and identified key immunosuppression-related genes, transcriptional regulatory factors and signaling pathways implicated in the molecular pathogenesis of ischemic stroke. The most prominent molecular mechanisms included 17 immunosuppression-related key crosstalk genes including ARG1, CD36, FCN1, GRN, IL7R, JAK2, MAFB, MMP9, PTEN, STAT3, STAT5A, THBS1, TLR2, TLR4, TLR7, TNFSF10, and VASP, transcription factors targeting STAT3, SPI1, CEPBD, SP1, TP53, NFIL3, STAT1, HIF1A, and JUN, and, enriched signaling pathways, PD-L1 expression and PD-1 checkpoint pathway, NF-kappa B signaling, IL-17 signaling, TNF signaling, and NOD-like receptor signaling.

## Data Availability Statement

The original contributions presented in the study are included in the article/Supplementary Material, further inquiries can be directed to the corresponding author/s.

## Author Contributions

XW: conceptualization, funding acquisition, methodology, formal analysis, and writing—original draft. QW, KW, and QN: methodology, formal analysis, and writing—original draft. HL and ZS: formal analysis, methodology and writing—review and editing. YX: project administration, supervision, and writing—review and editing. All authors contributed to the article and approved the submitted version.

## Conflict of Interest

The authors declare that the research was conducted in the absence of any commercial or financial relationships that could be construed as a potential conflict of interest.

## Publisher’s Note

All claims expressed in this article are solely those of the authors and do not necessarily represent those of their affiliated organizations, or those of the publisher, the editors and the reviewers. Any product that may be evaluated in this article, or claim that may be made by its manufacturer, is not guaranteed or endorsed by the publisher.

## Footnotes

1
http://www.ncbi.nlm.nih.gov/geo

2
http://www.disgenet.org

3
http://biokb.ncpsb.org.cn/HisgAtlas/

4
https://www.drugbank.ca/

5
http://thebiogrid.org/

6
https://mint.bio.uniroma2.it/

7
http://www.hprd.org/

8
https://www.ebi.ac.uk/intact/

9
http://ophid.utoronto.ca/ophidv2.204/

10
https://www.innatedb.ca/

11
https://www.grnpedia.org/trrust/

12
http://orti.sydney.edu.au/about.html

13
http://www.dgidb.org/search_interactions

## References

[B1] Alam J. J. Krakovsky M. Germann U. Levy A. (2020). Continuous administration of a p38α inhibitor during the subacute phase after transient ischemia-induced stroke in the rat promotes dose-dependent functional recovery accompanied by increase in brain BDNF protein level. *PLoS One* 15:e0233073. 10.1371/journal.pone.0233073 33275615 PMC7717516

[B2] Amantea D. Giacinto B. (2016). Drug repurposing for immune modulation in acute ischemic stroke. *Curr. Opin. Pharmacol.* 26 124–130. 10.1016/j.coph.2015.11.006 26657075

[B3] Ansar S. Eftekhari S. Waldsee R. Nilsson E. Nilsson O. Säveland H. (2013). MAPK signaling pathway regulates cerebrovascular receptor expression in human cerebral arteries. *BMC Neurosci.* 14:12. 10.1186/1471-2202-14-12 23343134 PMC3663811

[B4] Barr T. L. Conley Y. Ding J. Dillman A. Warach S. Singleton A. (2010). Genomic biomarkers and cellular pathways of ischemic stroke by RNA gene expression profiling. *Neurology* 75 1009–1014. 10.1212/wnl.0b013e3181f2b37f 20837969 PMC2942033

[B5] Busch H.-J. Schirmer S. H. Jost M. van Stijn S. Peters S. L. M. Piek J. J. (2010). Leptin augments cerebral hemodynamic reserve after three-vessel occlusion: distinct effects on cerebrovascular tone and proliferation in a nonlethal model of hypoperfused rat brain. *J. Cereb. Blood Flow Metab.* 31 1085–1092. 10.1038/jcbfm.2010.192 20978518 PMC3070967

[B6] Choi J.-S. Kim S. Y. Cha J.-H. Choi Y.-S. Sung K.-W. Oh S. T. (2003). Upregulation of gp130 and STAT3 activation in the rat hippocampus following transient forebrain ischemia. *Glia* 41 237–246. 10.1002/glia.10186 12528179

[B7] Diao X. Liu A. (2017). Identification of core pathways based on attractor and crosstalk in ischemic stroke. *Exp. Ther. Med.* 15 1520–1524. 10.3892/etm.2017.5563 29434737 PMC5776172

[B8] Fang W. Zhai X. Han D. Xiong X. Wang T. Zeng X. (2018). CCR2-dependent monocytes/macrophages exacerbate acute brain injury but promote functional recovery after ischemic stroke in mice. *Theranostics* 8 3530–3543. 10.7150/thno.24475 30026864 PMC6037034

[B9] Fu Y. Liu Q. Anrather J. Shi F.-D. (2015). Immune interventions in stroke. *Nat. Rev. Neurol.* 11 524–535. 10.1038/nrneurol.2015.144 26303850 PMC4851339

[B10] Garcia-Bonilla L. Faraco G. Moore J. Murphy M. Racchumi G. Srinivasan J. (2016). Spatio-temporal profile, phenotypic diversity, and fate of recruited monocytes into the post-ischemic brain. *J. Neuroinflammation* 13:285. 10.1186/s12974-016-0750-0 27814740 PMC5097435

[B11] Gelderblom M. Leypoldt F. Steinbach K. Behrens D. Choe C.-U. Siler D. A. (2009). Temporal and spatial dynamics of cerebral immune cell accumulation in stroke. *Stroke* 40 1849–1857. 10.1161/strokeaha.108.534503 19265055

[B12] Gomez-Sanchez J. C. Delgado-Esteban M. Rodriguez-Hernandez I. Sobrino T. Perez de la Ossa N. Reverte S. (2011). The human Tp53 Arg72Pro polymorphism explains different functional prognosis in stroke. *J. Exp. Med.* 208 429–437. 10.1084/jem.20101523 21357744 PMC3058581

[B13] Gu L. Xiong X. Wei D. Gao X. Krams S. Zhao H. (2013). T cells contribute to stroke-induced Lymphopenia in rats. *PLoS One* 8:e59602. 10.1371/journal.pone.0059602 23555048 PMC3598760

[B14] Gu Y. Wu Y. Chen L. (2021). GP6 promotes the development of cerebral ischemic stroke induced by atherosclerosis via the FYN-PKA-pPTK2/FAK1 signaling pathway. *Adv. Clin. Exp. Med.* 30 823–829. 10.17219/acem/135510 34418331

[B15] Gustavsen J. A. Pai S. Isserlin R. Demchak B. Pico A. R. (2019). RCy3: network biology using Cytoscape from within R. *F1000Res.* 8:1774. 10.12688/f1000research.20887.3 31819800 PMC6880260

[B16] Hu X. Li P. Guo Y. Wang H. Leak R. K. Chen S. (2012). Microglia/Macrophage polarization dynamics reveal novel mechanism of injury expansion after focal cerebral ischemia. *Stroke* 43 3063–3070. 10.1161/strokeaha.112.659656 22933588

[B17] Iadecola C. Anrather J. (2011). The immunology of stroke: from mechanisms to translation. *Nat. Med.* 17 796–808. 10.1038/nm.2399 21738161 PMC3137275

[B18] Iadecola C. Buckwalter M. S. Anrather J. (2020). Immune responses to stroke: mechanisms, modulation, and therapeutic potential. *J. Clin. Invest.* 130 2777–2788. 10.1172/jci135530 32391806 PMC7260029

[B19] Kaminska B. Gozdz A. Zawadzka M. Ellert-Miklaszewska A. Lipko M. (2009). MAPK signal transduction underlying brain inflammation and gliosis as therapeutic target. *Anat. Rec.* 292 1902–1913. 10.1002/ar.21047 19943344

[B20] Katan M. Luft A. (2018). Global burden of stroke. *Semin. Neurol.* 38 208–211. 10.1055/s-0038-1649503 29791947

[B21] Knox R. Jiang X. (2015). Fyn in neurodevelopment and ischemic brain injury. *Dev. Neurosci.* 37 311–320. 10.1159/000369995 25720756 PMC4713834

[B22] Krug T. Gabriel J. P. Taipa R. Fonseca B. V. Domingues-Montanari S. Fernandez-Cadenas I. (2012). TTC7B emerges as a novel risk factor for ischemic stroke through the convergence of several genome-wide approaches. *J. Cereb. Blood Flow Metab.* 32 1061–1072. 10.1038/jcbfm.2012.24 22453632 PMC3367223

[B23] Kursa M. B. (2014). Robustness of Random Forest-based gene selection methods. *BMC Bioinformatics* 15:8. 10.1186/1471-2105-15-8 24410865 PMC3897925

[B24] Kursa M. B. Rudnicki W. R. (2010). Feature selection with theBorutaPackage. *J. Stat. Softw.* 36 1–13. 10.18637/jss.v036.i11

[B25] Li Y. Prasad A. Jia Y. Roy S. G. Loison F. Mondal S. (2011). Pretreatment with phosphatase and tensin homolog deleted on chromosome 10 (PTEN) inhibitor SF1670 augments the efficacy of granulocyte transfusion in a clinically relevant mouse model. *Blood J. Am. Soc. Hematol.* 117 6702–6713. 10.1182/blood-2010-09-309864 21521784 PMC3123029

[B26] Liu F. Li L. Chen J. Wu Y. Cao Y. Zhong P. (2021). A network pharmacology to explore the mechanism of calculus bovis in the treatment of ischemic stroke. *Biomed Res. Int.* 2021:6611018. 10.1155/2021/6611 33778069 PMC7972848

[B27] Liu J. Zhou C.-X. Zhang Z.-J. Wang L.-Y. Jing Z.-W. Wang Z. (2012). Synergistic mechanism of gene expression and pathways between jasminoidin and ursodeoxycholic acid in treating focal cerebral ischemia-reperfusion injury. *CNS Neurosci. Ther.* 18 674–682. 10.1111/j.1755-5949.2012.00348.x 22726253 PMC6493368

[B28] Liu L. Doran S. Xu Y. Manwani B. Ritzel R. Benashski S. (2014). Inhibition of mitogen-activated protein kinase phosphatase-1 (MKP-1) increases experimental stroke injury. *Exp. Neurol.* 261 404–411. 10.1016/j.expneurol.2014.05.009 24842488 PMC10204778

[B29] Liu Q. Jin W.-N. Liu Y. Shi K. Sun H. Zhang F. (2017). Brain ischemia suppresses immunity in the periphery and brain via different neurogenic innervations. *Immunity* 46 474–487. 10.1016/j.immuni.2017.02.015 28314594

[B30] Luo H.-C. Yi T.-Z. Huang F.-G. Wei Y. Luo X.-P. Luo Q.-S. (2020). Role of long noncoding RNA MEG3/miR-378/GRB2 axis in neuronal autophagy and neurological functional impairment in ischemic stroke. *J. Biol. Chem.* 295 14125–14139. 10.1074/jbc.ra119.010946 32605923 PMC7549047

[B31] Mabuchi T. Kitagawa K. Ohtsuki T. Kuwabara K. Yagita Y. Yanagihara T. (2000). Contribution of microglia/macrophages to expansion of infarction and response of oligodendrocytes after focal cerebral ischemia in rats. *Stroke* 31 1735–1743. 10.1161/01.str.31.7.1735 10884481

[B32] Mao L. Jia J. Zhou X. Xiao Y. Wang Y. Mao X. (2013). Delayed administration of a PTEN inhibitor BPV improves functional recovery after experimental stroke. *Neuroscience* 231 272–281. 10.1016/j.neuroscience.2012.11.050 23219909 PMC3691271

[B33] Mo X. Li T. Ji G. Lu W. Hu Z. (2013). Peripheral polymorphonuclear leukocyte activation as a systemic inflammatory response in ischemic stroke. *Neurol. Sci.* 34 1509–1516. 10.1007/s10072-013-1447-0 23619532

[B34] Nagel S. Hadley G. Pfleger K. Grond-Ginsbach C. Buchan A. M. Wagner S. (2012). Suppression of the inflammatory response by diphenyleneiodonium after transient focal cerebral ischemia. *J. Neurochem.* 123 98–107. 10.1111/j.1471-4159.2012.07948.x 23050647

[B35] Newman A. M. Liu C. L. Green M. R. Gentles A. J. Feng W. Xu Y. (2015). Robust enumeration of cell subsets from tissue expression profiles. *Nat. Methods* 12 453–457. 10.1038/nmeth.3337 25822800 PMC4739640

[B36] Newman A. M. Steen C. B. Liu C. L. Gentles A. J. Chaudhuri A. A. Scherer F. (2019). Determining cell type abundance and expression from bulk tissues with digital cytometry. *Nat. Biotechnol.* 37 773–782. 10.1038/s41587-019-0114-2 31061481 PMC6610714

[B37] Pan Z. Ma G. Kong L. Du G. (2021). Hypoxia-inducible factor-1: regulatory mechanisms and drug development in stroke. *Pharmacol. Res.* 170:105742. 10.1016/j.phrs.2021.105742 34182129

[B38] Pedragosa J. Miró-Mur F. Otxoa-de-Amezaga A. Justicia C. Ruíz-Jaén F. Ponsaerts P. (2020). CCR2 deficiency in monocytes impairs angiogenesis and functional recovery after ischemic stroke in mice. *J. Cereb. Blood Flow Metab.* 40 S98–S116. 10.1177/0271678x20909055 32151226 PMC7687030

[B39] Planas A. M. Soriano M. A. Berruezo M. Justicia C. Estrada A. Pitarch S. (1996). Induction of Stat3, a signal transducer and transcription factor, in reactive microglia following transient focal cerebral ischaemia. *Eur. J. Neurosci.* 8 2612–2618. 10.1111/j.1460-9568.1996.tb01556.x 8996811

[B40] Proctor B. M. Ren J. Chen Z. Schneider J. G. Coleman T. Lupu T. S. (2007). Grb2 Is required for atherosclerotic lesion formation. *Arterioscler. Thromb. Vasc. Biol.* 27 1361–1367. 10.1161/atvbaha.106.134007 17363695

[B41] Rakers C. Schleif M. Blank N. Matušková H. Ulas T. Händler K. (2018). Stroke target identification guided by astrocyte transcriptome analysis. *Glia* 67 619–633. 10.1002/glia.23544 30585358

[B42] Ramos-Araque M. E. Rodriguez C. Vecino R. Cortijo Garcia E. de Lera Alfonso M. Sanchez Barba M. (2018). The neuronal ischemic tolerance is conditioned by the Tp53 Arg72Pro polymorphism. *Transl. Stroke Res.* 10 204–215. 10.1007/s12975-018-0631-1 29687302 PMC6421278

[B43] Sairanen T. Carpeìn O. Karjalainen-Lindsberg M.-L. Paetau A. Turpeinen U. Kaste M. (2001). Evolution of cerebral tumor necrosis factor-α production during human ischemic stroke. *Stroke* 32 1750–1758. 10.1161/01.str.32.8.1750 11486101

[B44] Satriotomo I. Bowen K. K. Vemuganti R. (2006). JAK2 and STAT3 activation contributes to neuronal damage following transient focal cerebral ischemia. *J. Neurochem.* 98 1353–1368. 10.1111/j.1471-4159.2006.04051.x 16923154

[B45] Sawe N. Steinberg G. Zhao H. (2008). Dual roles of the MAPK/ERK1/2 cell signaling pathway after stroke. *J. Neurosci. Res.* 86 1659–1669. 10.1002/jnr.21604 18189318

[B46] Schwartz D. M. Kanno Y. Villarino A. Ward M. Gadina M. O’Shea J. J. (2017). JAK inhibition as a therapeutic strategy for immune and inflammatory diseases. *Nat. Rev. Drug Discov.* 16 843–862. 10.1038/nrd.2017.201 29104284

[B47] Schwartz M. W. Baskin D. G. (2013). Leptin and the brain: then and now. *J. Clin. Invest.* 123 2344–2345. 10.1172/jci69346 23722910 PMC3668840

[B48] Shi K. Wood K. Shi F.-D. Wang X. Liu Q. (2018). Stroke-induced immunosuppression and poststroke infection. *Stroke Vasc. Neurol.* 3 34–41. 10.1136/svn-2017-000123 29600006 PMC5870641

[B49] Shichita T. Sugiyama Y. Ooboshi H. Sugimori H. Nakagawa R. Takada I. (2009). Pivotal role of cerebral interleukin-17–producing γδT cells in the delayed phase of ischemic brain injury. *Nat. Med.* 15 946–950. 10.1038/nm.1999 19648929

[B50] Shim R. Wong C. H. Y. (2016). Ischemia, immunosuppression and infection—tackling the predicaments of post-stroke complications. *Int. J. Mol. Sci.* 17:64. 10.3390/ijms17010064 26742037 PMC4730309

[B51] Stubbe T. Ebner F. Richter D. Engel O. R. Klehmet J. Royl G. (2012). Regulatory T cells accumulate and proliferate in the ischemic hemisphere for up to 30 days after MCAO. *J. Cereb. Blood Flow Metab.* 33 37–47. 10.1038/jcbfm.2012.128 22968321 PMC3597367

[B52] Sun J. Nan G. (2016). The mitogen-activated protein kinase (MAPK) signaling pathway as a discovery target in stroke. *J. Mol. Neurosci.* 59 90–98. 10.1007/s12031-016-0717-8 26842916

[B53] Takagi N. Cheung H. H. Bissoon N. Teves L. Wallace M. C. Gurd J. W. (1999). The Effect of transient global ischemia on the interaction of Src and Fyn with the N-Methyl-d-Aspartate receptor and postsynaptic densities: possible involvement of Src homology 2 domains. *J. Cereb. Blood Flow Metab.* 19 880–888. 10.1097/00004647-199908000-00007 10458595

[B54] Takenaga Y. Takagi N. Murotomi K. Tanonaka K. Takeo S. (2009). Inhibition of Src activity decreases tyrosine phosphorylation of occludin in brain capillaries and attenuates increase in permeability of the blood-brain barrier after transient focal cerebral ischemia. *J. Cereb. Blood Flow Metab.* 29 1099–1108. 10.1038/jcbfm.2009.30 19319148

[B55] Wei Y. Sun Z. Wang Y. Xie Z. Xu S. Xu Y. (2019). Methylation in the TP53 promoter is associated with ischemic stroke. *Mol. Med. Report* 20 1404–1410. 10.3892/mmr.2019.10348 31173230

[B56] Wong C. H. Y. Jenne C. N. Lee W.-Y. Léger C. Kubes P. (2011). Functional innervation of hepatic iNKT cells is immunosuppressive following stroke. *Science* 334 101–105. 10.1126/science.1210301 21921158

[B57] Wu Y. Xu J. Xu J. Zheng W. Chen Q. Jiao D. (2018). Study on the mechanism of JAK2/STAT3 signaling pathway-mediated inflammatory reaction after cerebral ischemia. *Mol. Med. Report* 17 5007–5012. 10.3892/mmr.2018.8477 29393445 PMC5865961

[B58] Yamauchi J. Miyamoto Y. Sanbe A. Tanoue A. (2006). JNK phosphorylation of paxillin, acting through the Rac1 and Cdc42 signaling cascade, mediates neurite extension in N1E-115 cells. *Exp. Cell Res.* 312 2954–2961. 10.1016/j.yexcr.2006.05.016 16814769

[B59] Yan J. Zhou B. Taheri S. Shi H. (2011). Differential effects of HIF-1 inhibition by YC-1 on the overall outcome and blood-brain barrier damage in a rat model of ischemic stroke. *PLoS One* 6:e27798. 10.1371/journal.pone.0027798 22110762 PMC3218033

[B60] Yilmaz G. Arumugam T. V. Stokes K. Y. Granger D. N. (2006). Role of T lymphocytes and interferon-γ in ischemic stroke. *Circulation* 113 2105–2112. 10.1161/circulationaha.105.593046 16636173

[B61] Yu G. Wang L.-G. Han Y. He Q.-Y. (2012). clusterProfiler: an R package for comparing biological themes among gene clusters. *OMICS* 16 284–287. 10.1089/omi.2011.0118 22455463 PMC3339379

[B62] Zhang L. Liu B. Han J. Wang T. Han L. (2020). Competing endogenous RNA network analysis for screening inflammation-related long non-coding RNAs for acute ischemic stroke. *Mol. Med. Rep.* 22 3081–3094. 10.3892/mmr.2020.11415 32945445 PMC7453507

